# Prostate MRI in the United Kingdom: a survey of current practice by the British Society of Urogenital Radiology

**DOI:** 10.1093/bjr/tqaf312

**Published:** 2025-12-14

**Authors:** Samuel J Withey, Iztok Caglic, Tristan Barrett

**Affiliations:** Department of Radiology, The Royal Marsden NHS Foundation Trust, London SW3 6JJ, United Kingdom; British Society of Urogenital Radiology, Devon, United Kingdom; British Society of Urogenital Radiology, Devon, United Kingdom; Centre for Medical Imaging, University College London, London WC1E 6AG, United Kingdom; British Society of Urogenital Radiology, Devon, United Kingdom; Department of Radiology, Addenbrookes Hospital, Cambridge University Hospitals NHS Foundation Trust, Cambridge CB2 0QQ, United Kingdom

**Keywords:** prostate MRI, prostate cancer, prostate biopsy, PSMA PET/CT, workforce, survey, biparametric, multiparametric

## Abstract

**Objectives:**

To characterize contemporary UK practice in prostate MRI reporting and pathways, and identify priorities for standardization.

**Methods:**

Between February and April 2025, a questionnaire was distributed by the British Society of Urogenital Radiology to UK-based consultant radiologists who report prostate MRI. It contained 34 questions, covering 6 domains: demographics; MRI protocols; biopsy; reporting preferences; imaging pathways; and attitudes towards accreditation.

**Results:**

Seventy-seven radiologists representing 52 NHS Trusts across all nations of the UK responded. Key findings include variable patient preparation for MRI including anti-spasmodic medication (69.2% administering) and instruction related to bowel preparation (13.5%). Trusts (100) were performing MRI prior to biopsy for suspected cancer; 73.1% using multiparametric MRI. When reporting prostate MRI for suspected cancer, 28.6% document only a PI-RADS score, 27.3% only a Likert score, with 44.1% stating both. The PI-QUAL score is moderately well known (71.4% of respondents) but not in routine use (7.8%). Transperineal prostate biopsy was performed at 88.5% of Trusts, with biopsy more likely to be performed by urologists (98.1% of Trusts) or urology nurses (36.5%) than radiologists (26.9%). Patients with high- or very high-risk prostate cancer undergo variable staging pathways, with university teaching hospitals more likely to offer PSMA PET/CT than other settings (33.3% vs 12.0%, *P = .*023).

**Conclusions:**

This survey shows the current state of UK prostate MRI practice, including universal pre-biopsy MRI. It identifies areas for standardization, including MRI protocols, scoring systems, and national staging guidelines.

**Advances in knowledge:**

Provides an up-to-date snapshot on prostate MRI within the UK.

## Introduction

Prostate cancer (PCa) is the most common malignancy affecting men in the UK, with incidence continuing to rise due to demographic changes and increased PSA testing.^1^ MRI has revolutionized the PCa pathway and is strongly recommended as the first diagnostic to allow MRI-directed biopsy of suspicious lesions, and for some men to safely avoid biopsy altogether.^2^^,^^3^

The MRI-directed prostate cancer diagnostic pathway is guided by the Prostate Imaging-Reporting and Data System (PI-RADS) v2.1, the 2019 National Institute for Health and Care Excellent (NICE) guideline, and 2025 update of the European Association of Urology (EAU) guidelines.^2^^–^^4^ PI-RADS v2.1 recommends a multiparametric MRI (mpMRI) protocol: axial and either coronal or sagittal T2-weighted images (or both), diffusion-weighted imaging (DWI), and dynamic contrast-enhanced fat-saturated T1-weighted imaging (DCE), stating that a biparametric protocol (bpMRI) without DCE should only be considered in select cases. The pelvic lymph nodes up to the aortic bifurcation should be covered by at least 1 sequence. PI-RADS comments that there is no consensus on patient preparation.^4^ Previous work, including the National Prostate Cancer Audit (NPCA) and 2019 Prostate Cancer UK (PCUK) survey, has highlighted heterogeneity of practice including provision of pre-biopsy MRI and differing MRI protocols.^5^^,^^6^

Since the 2019 PCUK survey, much has changed within the specialty, including a push for earlier diagnosis, interest in more streamlined protocols,^7^ shifts towards transperineal biopsy, widening active surveillance programmes, new scoring systems to improve accuracy and communication,^8–10^ and an interest in accreditation programmes.^11^ Reflecting this, in the UK, the genitourinary radiology workforce has grown more than other subspecialties over the past 5 years.^12^

The British Society of Urogenital Radiology (BSUR) is a Royal College of Radiologists-affiliated special interest group. One of its aims is to educate and improve standards in the field of urological radiology. Understanding variation in practice is critical to inform future national standards and to provide equitable care within the UK. Therefore, on behalf of the BSUR we have conducted this survey of UK-based consultant radiologists who report prostate MRI to comprehensively characterize current practice, and to identify areas for change.

## Methods

### Survey design and distribution

The survey was designed by 3 BSUR committee members (Authors S.J.W., I.C., T.B.), considered experts in prostate MRI.^13^ It comprised 34 questions, with a combination of multiple-choice questions, agreement levels with statements, and free text responses. The full list of questions is shown in Table S1. As this survey included no patient data, and respondents participated voluntarily with responses kept anonymous, institutional review board approval was not required.

An online survey was created on Microsoft Forms (Redmond, USA). An open call to participate was distributed by email to BSUR members, who in turn were encouraged to distribute the survey within their local cancer networks. To be eligible to participate, the respondents needed to (1) be practicing at consultant level in the UK, and (2) report prostate MRI in routine practice.

The survey comprised 6 major domains: (1) individual and centre demographics; (2) MRI protocols; (3) biopsy practice; (4) reporting preferences; (5) imaging pathways; and (6) attitudes towards accreditation. The survey was open for responses between February 19, 2025 and April 22, 2025.

### Statistical analysis

Quantitative responses were summarized with descriptive statistical analyses. Qualitative free-text comments were obtained for some questions, to allow deeper insight into radiologist attitudes. Subgroup analysis was performed by hospital type, using chi-square test.

## Results

The full results are shown in Table 1 (for individual radiologist-level data) and Table 2 (for hospital-level data).

**Table 1.** Survey answers for radiologist-level data.

About your hospital			
What category of hospital do you work at?			
University teaching hospital	*n = *43/77 (55.8%)		
Other	*n = *34/77 (44.2%)		
**Prostate MRI protocols**			
If using multiparametric MRI, would you support a change to biparametric MRI if recommended in guidance?			
Likely/highly likely would	*n = *11/59 (18.6%)		
Unsure	*n = *24/59 (40.7%)		
Likely/highly likely would not	*n = *24/59 (40.7%)		
**Suspected prostate cancer pathway**			
How is lesion location on MRI communicated to the person performing biopsy? (select all that apply)			
Annotations added to key images on PACS	*n = *49/77 (63.6%)		
Key series and images detailed in written report	*n = *46/77 (59.7%)		
Screenshots/key images saved	*n = *30/77 (39.0%)		
Described in report using the “clock face” position	*n = *28/77 (36.4%)		
Described in report using PI-RADS sector map	*n = *24/77 (31.2%)		
Diagram drawn by hand	*n = *7/77 (9.1%)		
Lesions fully segmented on MRI	*n = *5/77 (6.5%)		
Described in report in another way	*n = *4/77 (5.2%)		
Not applicable, biopsies only done by radiologist	*n = *1/77 (1.3%)		
**Individual experience**			
How many years of experience of prostate MRI reporting do you have art consultant level?			
1-2 years	*n = *11/77 (14.3%)		
3-5 years	*n = *13/77 (16.9%)		
6-9 years	*n = *14/77 (18.2%)		
10-15 years	*n = *22/77 (28.6%)		
More than 15 years	*n = *17/77 (22.1%)		
Estimate many prostate MRI studies you have reported in total			
<400	*n = *5/77 (6.5%)		
400-1000	*n = *20/77 (26.0%)		
>1000	*n = *52/77 (67.5%)		
Estimate how many prostate MRI studies you have reported in the past year			
Median (IQR)	300 (200-475)		
Range	50-1500		
Have you attended a prostate MRI course in the past 3 years?			
Yes	*n = *46/77 (59.7%)		
No	*n = *31/77 (40.3%)		
How many prostate MRI courses have you attended in total (including as faculty)?			
0	*n = *4/77 (5.2%)		
1	*n = *26/77 (33.8%)		
2	*n = *16/77 (20.8%)		
3	*n = *16/77 (20.8%)		
4 or more	*n = *15/77 (19.5%)		
** Rate your level of agreement with the following statements:**	**Strongly Disagree or Disagree**	**Neutral**	**Strongly Agree or Agree**
Formal accreditation is necessary to ensure high quality prostate MRI reporting	*n = *19/77 (24.7%)	*n = *29/77 (37.7%)	*n = *29/77 (37.7%)
I currently have plans to gain formal accreditation	*n = *45/77 (58.5%)	*n = *22/77 (28.6%)	*n = *10/77 (13.0%)
If accreditation were to become mandatory, then I would look to gain accreditation	*n = *59/77 (76.6%)	*n = *9/77 (11.7%)	*n = *9/77 (11.7%)
**Prostate MRI reporting**			
Do you give a PI-RADS or Likert score in the setting of suspected prostate cancer?			
PI-RADS only	*n = *22/77 (28.6%)		
Likert only	*n = *21/77 (27.3%)		
Both	*n = *34/77 (44.2%)		
If giving a PI-RADS score, do you let clinical factors influence this?			
Yes	*n = *20/56 (35.7%)		
No	*n = *36/56 (64.3%)		
**Which of the following scoring systems are using aware of? And using in routine practice?**	**Not aware of**	**Aware of but not using**	**Aware of and using routinely**
PI-RADS	*n = *0/77 (0%)	*n = *19/77 (24.7%)	*n = *58/77 (75.3%)
PI-QUAL	*n = *22/77 (28.6%)	*n = *49/77 (63.6%)	*n = *6/77 (7.8%)
PRECISE	*n = *24/77 (31.2%)	*n = *35/77 (45.5%)	*n = *18/77 (23.4%)
PI-FAB	*n = *58/77 (75.3%)	*n = *17/77 (22.1%)	*n = *2/77 (2.6%)
PI-RR	*n = *52/77 (67.5%)	*n = *22/77 (28.6%)	*n = *3/77 (3.9%%)

**Table 2.** Survey answers for hospital-level data.

About your hospital	
What category of hospital is this?	
University teaching hospital	*n = *25/52 (48.1%)
Other	*n = *27/52 (51.9%)
How many radiologists report prostate MRI at your hospital?	
2	*n = *8/52 (15.4%)
3	*n = *6/52 (11.5%)
4-6	*n = *28/52 (53.8%)
7-9	*n = *8/52 (15.4%)
10 or more	*n = *2/52 (3.8%)
How many radiologists are regularly involved in a prostate MDT meeting at your hospital?	
1	*n = *2/52 (3.8%)
2	*n = *12/52 (23.1%)
3-4	*n = *27/52 (51.9%)
5-6	*n = *5/52 (9.6%)
7-9	*n = *6/52 (11.5%)
Which Radiology Information System (RIS) codes are used for prostate MRI at your hospital? (select all that apply)	
MPEST/MPESTC	*n = *28/52 (53.8%)
MPROS/MPROSC	*n = *15/52 (28.8%)
MPELV/MPELVC	*n = *11/52 (21.2%)
MPLPVC	*n = *1/52 (1.9%)
MMPMP	*n = *9/52 (17.3%)
**Prostate MRI protocols**	
For suspected localized prostate cancer, do you use biparametric or multiparametric MRI as standard?	
Multiparametric MRI	*n = *38/52 (73.1%)
Biparametric MRI	*n = *14/52 (26.9%)
In addition to the sequences required for prostate MRI, do you perform any additional sequences?	
Large field of view T1w pelvis	*n = *48/52 (92.3%)
Upper abdominal imaging	*n = *7 (13.5%)
Additional Pelvic DWI with different field of view	*n = *5/52 (9.6%)
Other sequences of pelvis	*n = *3/52 (5.8%)
Sagittal T2w lumbar spine	*n = *1/52 (1.9%)
No additional sequences	*n = *4/52 (7.7%)
What patient preparation steps are taken routinely for prostate MRI?	
Empty bowel prior to scan	*n = *7/52 (13.5%)
Bowel prep/enema administration	*n = *0/52 (0.0%)
Low residue diet in preceding days	*n = *0/52 (0.0%)
Refrain from ejaculation in preceding days	*n = *7/52 (13.5%)
Buscopan administration (intravenous)	*n = *27/52 (51.9%)
Buscopan administration (intramuscular)	*n = *6/52 (11.5%)
Buscopan administration (sublingual)	*n = *1/52 (1.9%)
Glucagon administration (intramuscular)	*n = *1/52 (1.9%)
Mebeverine administration (oral)	*n = *1/52 (1.9%)
**Suspected prostate cancer pathway**	
In the setting of suspected prostate cancer, which is performed first, MRI or biopsy?	
MRI	*n = *52/52 (100%)
Biopsy	*n = *0/52 (0.0%)
Which biopsy technique is used routinely? (select all that apply)	
Transperineal biopsy (under local anaesthetic)	*n = *44/52 (84.6%)
Transperineal biopsy (under general anaesthetic)	*n = *10/52 (19.2%)
Transrectal biopsy	*n = *10/52 (19.2%)
How are biopsy cores targeted towards lesions on MRI?	
Cognitive targeting	*n = *43/52 (82.7%)
MRI-US fusion targeting	*n = *8/52 (15.4%)
In-bore MRI biopsy	*n = *0/52 (0.0%)
No targeted biopsies taken	*n = *1/52 (1.9%)
Who performs prostate biopsy at your Trust?	
Urologists	*n = *51/52 (98.1%)
Clinical nurse specialists	*n = *19/52 (36.5%)
Radiologists	*n = *14/52 (26.9%)
Radiographers	*n = *3/52 (5.8%)
Are cases with positive MRI but negative biopsies discussed in an MDT/post-biopsy meeting?	
Yes	*n = *49/52 (94.2%)
No	*n = *1/52 (1.9%)
Not sure	*n = *2/52 (3.8%)
In high or very high-risk prostate cancer, what would be your typical staging investigation?	
CT and bone scintigraphy	*n = *37/52 (71.2%)
CT only	*n = *1/52 (1.9%)
Bone scintigraphy only	*n = *5/52 (9.6%)
PSMA PET/CT	*n = *9/52 (17.3%)
Whole body MRI	*n = *0/52 (0.0%)

### Demographics

**Figure 1.**
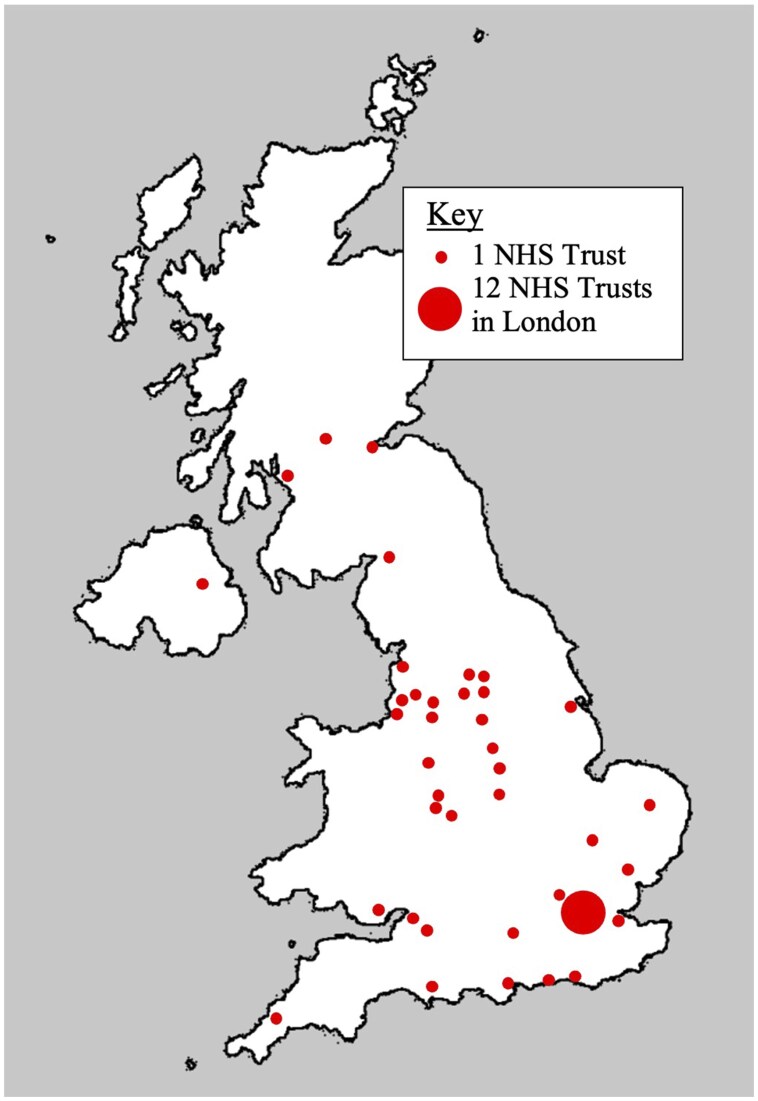
A map of the United Kingdom showing the location of NHS Trusts represented in this survey.

Seventy-seven radiologists responded to the survey, representing 52 NHS Trusts and 1 teleradiology provider. There were responders from all 4 nations of the UK; 47 of 52 Trusts (90.3%) were in England (Figure 1). Respondents (44/77; 57.1%) described their main employment setting as a university teaching hospital. Fifty-two radiologists (67.5%) were considered experts, having reported >1000 studies.^13^ Respondents were asked to estimate the number of prostate MRI studies they report per year (excluding multidisciplinary team meeting [MDT] reviews and second opinions). The range was 30 to 1500, median 300.

**Figure 2.**
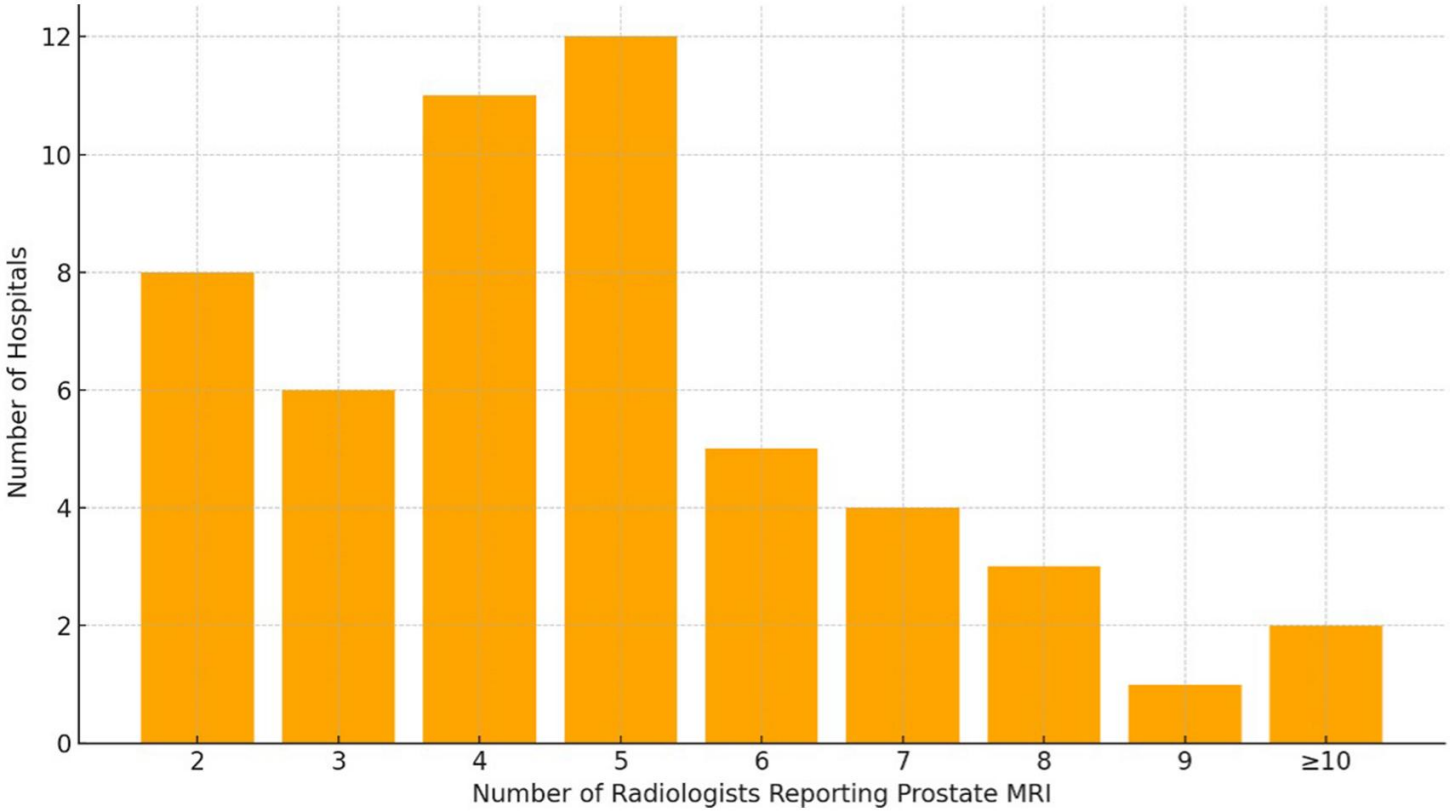
Distribution of the number of radiologists reporting prostate MRI per Trust.

The median number of prostate MRI reporters at each Trust was 5 (Figure 2). Twenty-eight of 52 (53.8%) Trusts had radiologists reporting prostate MRI who were not regularly involved in a prostate MDT. One Trust had a radiologist participating in the MDT, who did not routinely report prostate MRI.

### MRI protocols

Patients were given varying preparation before prostate MRI. At 7 Trusts (13.5%), patients were advised to empty their bowel immediately prior to the scan, and at 7 Trusts, patients were advised to refrain from ejaculation in the days preceding the MRI scan. Anti-spasmodics were used routinely at 69.2% of Trusts (*n = *36/52), including Buscopan (hyoscine butylbromide) administered intravenously (*n = *27, 51.9%), via intramuscular injection (*n = *6, 11.5%), or sublingually (*n = *1, 1.9%), intramuscular glucagon (*n = *1, 1.9%), and oral mebeverine (*n = *1, 1.9%). Fourteen Trusts (26.9%) did not routinely administer medication or provide preparation instructions.

In the setting of suspected localized PCa, 14/52 (26.9%) of Trusts perform bpMRI, 38/52 (73.1%) mpMRI. There was no significant difference in bpMRI versus mpMRI use between university teaching hospitals and other settings (bpMRI, 28.0% vs 25.9%; *P* = 1.0).

The 59 radiologists using mpMRI as standard were asked whether they would change practice to follow a bpMRI protocol if this was recommended in updated guidelines. Eleven (18.6%) said they were either unlikely or highly unlikely to change to bpMRI; 24 (40.7%) said the shift was likely or highly likely; with the remainder unsure. Free text answers were collected to gather opinions on mpMRI versus bpMRI (Supplementary Material). Key themes included efficiency and cost savings; and differing opinions on when DCE is considered helpful.

Most Trusts incorporated prostate MRI protocols with additional sequences beyond those considered mandatory by PI-RADS.^4^  *n = *7 (13.5%) routinely performed upper abdominal imaging, *n = *5 (9.6%) performed additional DWI sequences with a different field-of-view (ie, whole pelvis), *n = *3 performed other pelvic sequences (5.8%), and 1 performed sagittal T2w of the lumbar spine.

### Reporting preferences

Respondents were asked which scoring system they used to communicate the likelihood of clinically significant PCa. A PI-RADS score only was used by 22/77 (28.6%), Likert score only by 21/77 (27.3%), and both were given by 34/77 (44.1%). Notably, for respondents giving a PI-RADS score, 20/56 (35.7%) stated they would let clinical factors affect their PI-RADS score.

The 76 responders who worked in Trusts where biopsy was performed by non-radiologists were asked how lesion location was communicated to the individual performing biopsy. More than one method could be used by each reporter. The most common methods were annotation of images on PACS (*n = *49) and reference to key images detailed in the report (*n = *46). Lesion location was described using the “clock face” technique by 28 and using the PI-RADS sector map by 24. Diagrams were drawn by 7, and the lesions were fully segmented on MRI by 7.

**Figure 3.**
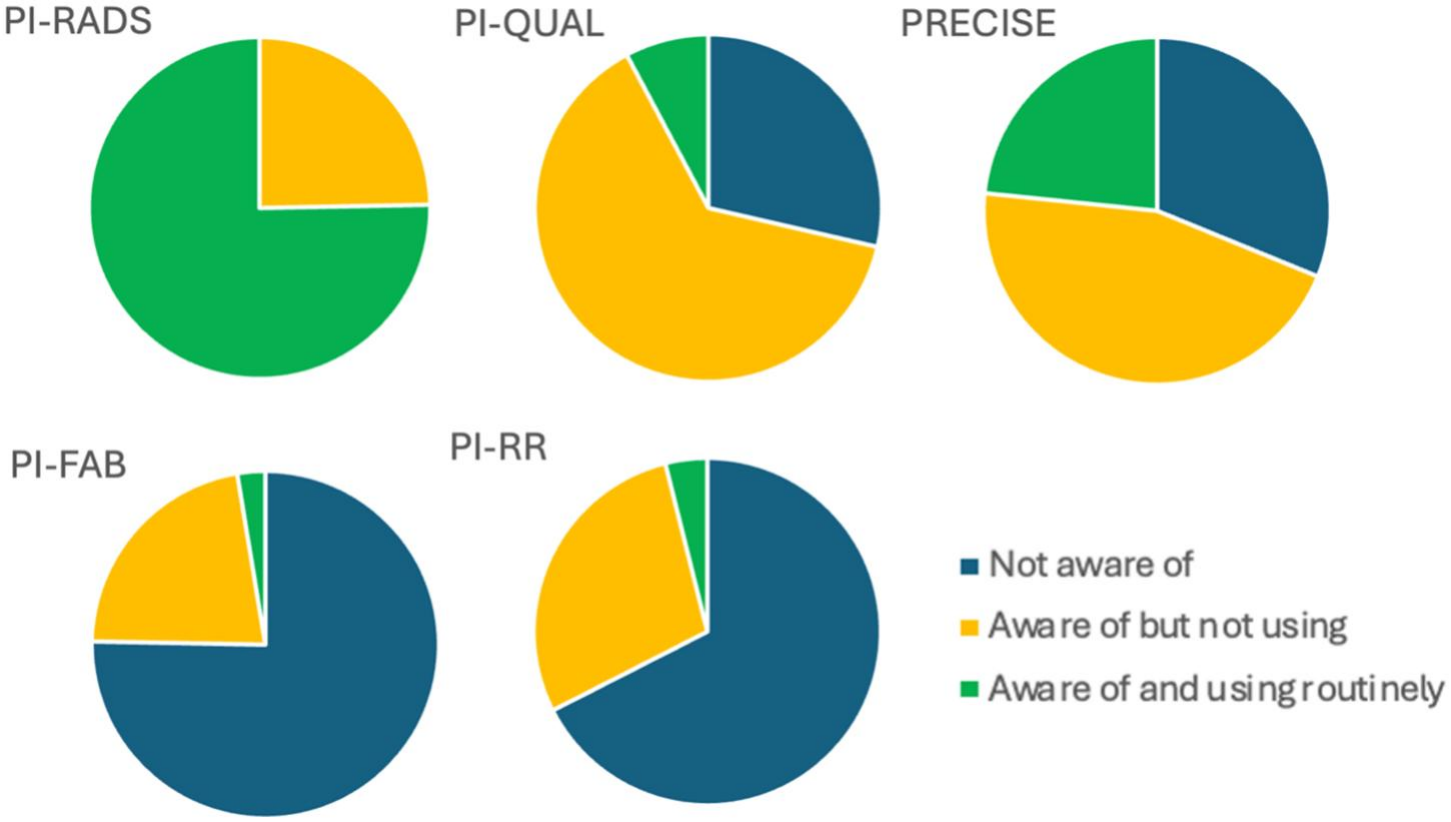
Distribution of responses regarding knowledge and use of different prostate MRI scoring systems.

There are several additional scoring systems that can be used in prostate MRI; radiologists were asked whether they were aware of these scoring systems and/or using them in routine practice (Figure 3). Other scoring systems were less used and less well known than PI-RADS or Likert: PI-QUAL (Prostate Imaging Quality scoring system) was known by 71.4% but only used by 7.8%^8^; PRECISE (Prostate Cancer Radiological Estimation of Change in Sequential Evaluation scoring system for use in active surveillance), PI-FAB (Prostate Imaging after Focal ABlation) and PI-RR (Prostate MRI for local Recurrence Reporting) were used by 23.4%, 2.6%, and 3.9%, respectively.^9^^,^^10^^,^^14^ Free text responses related to the scoring systems (Supplementary Materials) included the following themes: use of multiple systems can lead to confusion; PI-QUAL is a useful quality assurance tool but unnecessary in day-to-day reporting. Respondents who work in university teaching hospitals were more likely to be using or be aware of PRECISE, PI-FAB, and PI-RR (*P = .*005, *P = .*045, and *P = .*013, respectively). The difference was not significant for PI-QUAL (*P = .*092). Participants were not specifically asked which version of these scoring systems they used.

### Biopsy

Prostate MRI is performed before biopsy routinely in all 52 Trusts surveyed. Forty-six of 52 Trusts (88.5%) perform transperineal biopsy, with 44/46 (95.7%) offering this under local anaesthetic. Fifty-one of 52 Trusts (98.1%) are performing target biopsies as standard (15.4% using MRI-ultrasound fusion, 82.7% using cognitive fusion). Biopsies are most commonly performed by urologists (51/52 Trusts, 98.1%), but also by urology clinical nurse specialists (19/52, 36.5%), radiologists (14/52, 26.9%), and radiographers (3/52, 5.8%).

### Imaging pathways

In patients who have a high probability MRI (PI-RADS or Likert 4-5) but subsequent negative biopsy, NICE guidelines recommend MDT discussion. This was routine practice in 49/52 Trusts (94.2%), not standard practice in 1/52, with 2 respondents unsure.

Radiologists were asked about typical staging pathways in their Trusts. In patients with high or very high risk PCa (defined according to the Cambridge prognostic groups as any of: definite cT3-T4 stage, Gleason score 8-10, or PSA >20.0) what would be your typical staging investigation^15^: prostate specific membrane antigen (PSMA)-PET/CT was used in 9/52 (17.3%), CT with bone scintigraphy in 37/52 (71.2%), bone scan alone in 5/52 (9.6%) and CT alone in 1 (1.9%). University teaching hospitals were significantly more likely to offer PSMA PET/CT in this scenario (33.3% versus 12.0%, *P = .*023). No Trusts included in the survey were routinely performing whole body MRI for high-risk staging.

### Attitudes towards accreditation

Forty-six of 77 respondents had attended a prostate MRI course in the preceding 3 years (either as a delegate or faculty); 4/77 had never attended a prostate MRI course. Respondents were asked how much they agreed with 3 statements on accreditation:

1. Formal accreditation is necessary to ensure high quality prostate MRI reporting: 37.7% either agreed or strongly agree; 24.7% either disagreed or strongly disagreed; the remainder were neutral.
2. I currently have plans to gain formal prostate MRI accreditation: 13.0% agree/strongly agree; 58.5% disagree/strongly disagree.
3. If formal accreditation were to become mandatory, then I would look to gain it: 76.6% agree/strongly agree; 11.7% disagree/strongly disagree.

## Discussion

This survey of prostate MRI in the UK incorporates responses from 77 consultant radiologists representing 52 NHS Trusts and updates similar work from 2019.^6^ We detail areas of consistent practice, including ubiquitous use of a numerical score to convey risk of PCa, 100% of included NHS Trusts offering pre-biopsy MRI compared to 86% in 2019, and high availability of transperineal biopsy under local anaesthetic. In addition, we highlight areas of variation, such as within MRI protocols and patient preparation, imaging for staging high-risk disease, and individual attitudes towards accreditation.

### Demographics

Although no Trust had a single prostate reporter, 8 had only 2 radiologists able to report prostate MRI, which could pose challenges to service delivery during period of leave. The 2020 UK consensus meeting on prostate MRI certification reached agreement that to report independently, a radiologist should be attending at least 12 MDT meetings per year,^11^ however, 28/52 Trusts had at least one radiologist reporting who did not attend the prostate MDT.

### MRI protocols

There was significant variation in patient preparation for prostate MRI, which is not explicitly recommended in the current PI-RADS guidelines. Different instructions relating to bowel preparation and ejaculation mirror the literature, which has shown mixed results. Most studies do show some benefit for anti-spasmodic administration,^16^^,^^17^ and in our survey anti-spasmodics were administered in most Trusts, most commonly Buscopan (either by intravenous or intramuscular injection). Future work should assess the effect of pharmacological and non-pharmacological preparation on MRI quality. This is an area for potential improvement in quality and efficiency.

There is debate around the need for contrast administration in prostate MRI. When applying PI-RADS v2.1 strictly, it has only a limited role in upgrading a PI-RADS score of 3 to 4 in the peripheral zone, or as the secondary sequence when DWI is suboptimal. Although PI-RADS and the NICE guidelines recommend mpMRI, 26.9% of Trusts are performing bpMRI for suspected PCa, down from 34.9% in 2019. Respondents currently performing mpMRI as standard were polarized regarding their attitudes to DCE (Supplementary Materials): some felt strongly that it rarely informs interpretation; others felt that it frequently does and highlighted individual cases where significant cancer was only visible on DCE. Several respondents cited problems with image quality, particularly DWI, necessitating reliance on DCE at their centres. Several studies have shown non-inferiority of bpMRI compared to mpMRI, although it may offer benefit to less experienced readers.^18^^,^^19^ Since the survey closed to responses, the prospective PRIME trial has demonstrated bpMRI is non-inferior to mpMRI at identifying clinically significant PCa when image quality is adequate.^7^ Overall, opinions on the need for DCE seemed balanced, and with an update to the NICE guideline on prostate cancer due it will be interesting to see what the guidelines recommend, and whether they are adhered to.

Whilst a change to a bpMRI could lead to efficiency savings, including reduced scan duration, many Trusts are routinely performing additional MRI sequences beyond those mandated by PI-RADS v2.1, including upper abdominal imaging, additional large field-of-view DWI, other pelvic imaging eg, STIR, and sagittal T2w of the lumbar spine. Upper abdominal and lumbar spine imaging are difficult to justify given the limited clinical relevance, additional time taken to perform and report, and ongoing burden from incidental findings.

### Biopsy

In the current survey, all 52 Trusts were offering pre-biopsy MRI, versus 86% in 2019,^6^ and compared to a reported 35.5% in the USA.^20^

Level 1 evidence has shown that transperineal biopsy outperforms transrectal biopsy in diagnosis of PCa and is now recommended by EAU guidance.^3^^,^^21^ In the current survey, 88.5% of Trusts perform transperineal biopsy, with the vast majority offering this under local anaesthetic. Only a single Trust had only radiologists performing biopsy. 36.5% have nurses undertaking biopsy, and 5.8% radiographers, reflecting the wider shift towards extended roles for allied healthcare professionals within the NHS.

### Reporting preferences

Scoring systems have been introduced for prostate MRI reporting to standardize interpretation and improve communication between radiologists and clinicians. In the setting of suspected PCa, PI-RADS is the dominant scoring system used worldwide. However in the UK, NICE guidelines, written after publication of the PROMIS trial,^22^ recommended using a Likert score which allows more flexibility than PI-RADS and may take clinical factors into account. In our survey, 28.6% of radiologists are giving a PI-RADS score only, 27.3% are giving a Likert score only, and the remainder are giving both scores in their reports. The widespread use of 2 similar but different 5-point scoring systems to convey the same clinical risk has potential to reduce clarity in communication, contrary to the aim of categorical scoring systems. Interestingly, 20/56 respondents using PI-RADS allow their scoring to be affected by clinical factors, against the PI-RADS guidance. Moreover, biopsy decision aids proposed by the EAU consider PI-RADS score and PSA density, therefore applying a clinical weighting when assigning a PI-RADS score, eg, upgrading from 2 to 3 because of high PSA, runs the risk of the clinical factor being “double counted” in clinical decision-making.

The PI-QUAL scoring system evaluates prostate MR image quality.^8^ More than two-thirds of respondents are aware of the system, yet only 11.5% apply this in their clinical practice. Respondents felt that while useful as a quality-assurance tool, PI-QUAL is time-consuming and often the reason for suboptimal imaging cannot be addressed, eg, hip arthroplasty. Many respondents do comment on image quality, particularly for suboptimal studies, but base this on almost instant subjective assessment rather than a formal PI-QUAL score.

The PRECISE scoring system was known by 68.8% of respondents but used routinely by less than a quarter. The main reasons for low uptake included (1) use of too many 5-point scoring systems in reports may cause confusion, (2) a feeling it offers no benefit over a descriptive report, and (3) concern that many of the recommendations lack an evidence base, for instance 50% lesion volume increase indicating “radiological progression” in PRECISE v2.^14^

### Imaging pathways

For patients with high or very high-risk PCa, distant staging is recommended.^3^^,^^15^ Our survey highlights variable practice: most perform CT with bone scintigraphy (71.2% of Trusts), however, 17.3% of Trusts are performing PSMA PET/CT in this setting. Only in the 2025 update to the EAU guidelines was there a strong recommendation to perform PSMA PET/CT in high and very high-risk disease, and a weak recommendation for PET/CT in intermediate risk patients with Gleason Grade Group 3.^3^ It was widely acknowledged that PSMA PET/CT has superior diagnostic accuracy, but respondents highlighted 2 key access issues: first, PET/CT scanner availability varies by region; second, even in centres with a scanner, local policies governing its use are inconsistent. Notably, university teaching hospitals were significantly more likely to perform PSMA PET/CT. In addition, treatment paradigms are based on conventional staging investigations—there is limited evidence regarding the management of patients with metastatic disease which is only visible on next generation imaging.^23^^,^^24^

### Attitudes towards accreditation

The prostate diagnostic pathway is heavily reliant on an individual radiologist’s interpretation. As a result, there is interest in prostate MRI accreditation to help drive standards.^11^ Respondents (37.7%) thought that formal accreditation is necessary to ensure high quality reporting; 24.7% disagreed. Respondents (11.7%) respondents said that if accreditation were to become mandatory, they would stop reporting prostate MRI. The 2020 UK consensus meeting on accreditation proposed that for independent reporting, individuals should regularly participate in MDTs, attend 3-yearly prostate MRI course attendance, and obtain a minimum number of CPD/CME points related to prostate MRI, with some debate regarding an examination.^11^ Our survey implies the majority are fulfilling most of these criteria already.

## Conclusion

This survey of UK-based consultant radiologists incorporates 52 Trusts across all 4 nations of the UK and highlights areas of progress since the 2019 Prostate Cancer UK survey,^6^ including now universal adoption of pre-biopsy MRI. We identify potential areas for standardization, including MR imaging protocols, use of scoring systems, and national staging guidelines.

## Supplementary Material

tqaf312_Supplementary_Data
