# Supplementary material for: Prostate MRI in the United Kingdom: a survey of current practice by the British Society of Urogenital Radiology
Source: Br J Radiol. 2025 Dec 14;99(1179):442–9. doi: 10.1093/bjr/tqaf312 (PMC13017365; doi:10.1093/bjr/tqaf312)
Supplement: tqaf312_Supplementary_Data [file tqaf312_supplementary_data.docx]

**Supplementary material**

**Free text responses to the question: Please give any additional comments related to using biparametric versus multiparametric MRI for suspected prostate cancer**

- Follow guideline to be compliant for trials
- Save time and money
- Do worry about missing some cancers, visible on DCE but not DWI/T2w"
- Used bpMRI before merger. Think contrast rarely helps when scanners are optimised personally. Just a nice safety net
- Quicker, cheaper, better for patients and service
- Contrast helpful if poor quality for any reason…. On well optimised protocol generally not used that much. Although we do like looking at the prolonged contrast enhancement in inflammatory change
- Still find DCE useful for some cases, and weening off is challenging. A more explicit guidance on when to use/not (or when to employee recalls) would help
- I have too many significant cancers in my teaching library only detected by DCE. People get too hung up about detecting cancer, it is equally important we are safely excluding significant cancer so we can safely discharge back to primary care for PSA follow-up
- We’re involved in the Pacific study which aims to also look at benefit of using a biparametric protocol
- Contrast is so useful for lesion conspicuity (first and foremost), problem solving, and as a backstop for artefact on the other sequences (most of which is unpredictable). This seems to escape those who say it's not necessary because it plays a small role in PI-RADS and they just biopsy all their 3s anyway. I suspect they have never had the chance to use it in regular practice, so don't know what they are missing. I wouldn't be without it. Therefore I wouldn't want to see guidelines change as then we might be forced to drop contrast, which would certainly reduce diagnostic accuracy in my and most of my colleagues’ hands. In fact I'd rather drop MRI prostate reporting altogether than do bpMRI as standard
- Would expect an evidence-based decision
- The DCE sequences allow us to confirm presence of pathology, non-malignant conditions and convert a potentially inadequate exam (e.g. when DWI/ADC fails because of THR etc) into a diagnostic exam
- Our B1400 images are not of great quality, so we do find contrast quite helpful in equivocal cases. If we manage to consistently get good quality DWI, we may not need contrast
- While dynamic contrast enhancement offers some increased sensitivity it is my experience that this is not relevant in the majority of cases. Please await the results of the Pacific trial
- Supplementary information [from DCE] adds value to the study in complex cases
- We already do bpMRI in men > 70 [years old] with higher PSA values, and as part of active surveillance
- Dynamic contrast-enhanced study is still of value for patients with equivocal findings
- It would not be a decision I would make alone and would need to be a decision made as a reporting group based on the evidence presented.
- According to my experience, bpMRI prostate might give answer to clinical questions in most of patients and will save time and valuable resources
- In the event of compromised or degraded DWI/ADC, the DCE images are of great help.
- I like gadolinium but in reality it probably doesn't change assessment in most cases
- Contrast can be useful in some cases but opinion is mixed in our department.
- With national adopted guidelines, yes. Current literature suggest bpMRI is non-inferior. An internal audit I conducted years ago also suggested the same
- I would generally be in favour of bpMRI, but there are differing opinions within the department
- The contrast often helps in equivocal cases in the first diagnostic scan. For follow-up, would more likely consider doing as bpMRI
- We find contrast useful so might continue with it
- Still find contrast useful in some scenarios
- Gadolinium helps in accurate PI-RADS assessment. A few subtle lesions become more obvious on Gadolinium sequences.
- Depends on scan quality. Bad scans are often saved by T1 post contrast
- Multiple examples of cases where the presence of contrast helped with detection of lesions and therefore significantly alters management.
- Helps characterise lesions that are otherwise indeterminate
- Pressure on service would drive quicker scans, also less environmental impact
- PI-RADS is based on mpMRI – the DCE sequences matter!
- Although sometimes helpful, I feel like DCE helps me better characterise a prostrate abnormality in an actionable way no more than 10% of the time (I.e. biopsy vs no biopsy), and occasionally it can help with establishing early T3b disease, and sometimes with early involvement of the membranous urethra. I feel like it potentially confounds my report more often though, by compelling me to declare a nonspecific (or probably benign) area of mild restricted diffusion in the PZ as PI-RADS 4 (by mandating an upgrade of a soft PI-RADS 3). Too many prostates are reported as PI-RADS 4 for this reason and are over investigated. And so this not infrequently overstates or indeed misrepresents my actual opinion. For this reason I find I sometimes report something as non-specific/favoured benign (Likert 2/ PIRADS 4), which is an obviously confusing message to the clinician. For the added time needed to cannulate the patient, do the additional sequences, read them, and the risks of contrast allergy (especially outside of normal working hours), it is too low yielding and not worth it.
- I don't find contrast that helpful
- I understand from PRIME, bpMRI is not inferior but I think the overall quality has to be improved across the country for this to be recommended
- I think the argument to give contrast is reducing, and in the context of quicker scan times, deep resolve sequences etc, one could argue that DCE is not necessary anymore
- Before we started mpMRI I did not feel we needed to use contrast but since we started using it, we got new magnets and despite excellent MR physics support the images from the new machines are not as reliable and I can't 'get my eye in' to our new images. Contrast really helps me to decide about lesions. I would not be without it now.
- In most cases, bpMRI is enough.
- For straight-to-test or initial exam. Would prefer contrast for cases where they have had previous treatment (e.g. HIFU/radiotherapy) or for trouble shooting.
- DCE often useful
- mpMRI as standard, bpMRI for PSA <5 or >30 and for all active surveillance patients
- Depends on the evidence and the guidelines
- I think the efficiency savings [of bpMRI] are worth it
- Evidence and personal experience shows that DCE is a more reliable sequence than DWI (in a real-world setting)
- It really depends on the quality of T2WI and DWI
- Old MRI scanner with rubbish DWI
- Currently use mpMRI protocol to comply with PI-RADS v2 (so DCE needed). If DCE no longer mandated, then would consider removing this sequence however, DCE is useful in cases where rectal gas artefact compromised DWI. Removing DCE might necessitate patient recall if DWI compromised (as no DCE to use as a secondary sequence). Would need to assess impact of removing DCE on recall rate (currently extremely low).
- I appreciate the push to ditch contrast images due to time and capacity. I find useful in some scenarios
- I think the evidence shows that the addition of a contrast enhanced sequence does not significantly change the detection of clinically significant prostate cancer. Removing it would improve efficiency without significantly changing diagnostic accuracy.
- Rarely find contrast essential, could be given on an ad hoc basis for unclear cases which would save money.
- Depends on the data, e.g. from the PACIFIC study
- Await results of pacific trial
- Dropping the DCE sequences would save time
- bpMRI would potentially allow patients to be scanned at weekends and in remote locations (without radiologist cover)
- Personally, I don't find DCE too helpful and feel that it results in over calling of P4 lesions
- Not necessarily for patients in all age/risk groups.
- Large volume of prostate MR locally, all pre-biopsy. bpMRI would result in reduction in scan length and cost savings.
- bpMRI detects majority of the aggressive malignancies, and we could use DCE for select group of patients. Also DCE only helps in the additional 10-15 % of cancer detection
- We use mpMRI as a 1st scan only, with all subsequent being bpMRI.
- As a group I think we are quite confident with bpMRI, it would be time and cost saving.
- Contrast still useful for a great many cases

**Free text responses to the question: Please give any additional comments related to imaging used for distant staging of high / very high-risk prostate cancer.**

- No PSMA PET CT service locally
- Depends upon treatment options
- Transition to PSMA PET instead of bone scan
- Would like to start WB MRI for selected cohorts
- CT TAP also performed [in addition to bone scan] if patient is candidate for novel hormones or has very high PSA (>100)
- Would prefer PSMA PET if readily available
- PSMA PET used where possible (and where guidelines allow) but sometimes poor local access
- No set protocol for distant staging – varies between clinicians
- Would do more PSMA PET if available
- [whole body MRI] Diffusion skeletal imaging should be used more widely
- PSMA PET should be used for high-risk non-metastatic disease and known metastases – we are still using bone scans and CT like 40 years ago
- PSMA PET used to assess equivocal findings
- Isotope bone scans should be viewed as obsolete – PSMA PET or no distant staging unless it would alter management
- PSMA PET sometimes overused for low-risk patients – should be ratified at MDT first
- PSMA PET can cause incidental findings of no importance, leading to dilemmas
- PSMA PET performed for high-risk patients if initial staging is negative
- Even with negative CT TAP and no bone metastases on mpMRI, some clinicians still want bone scan
- Depends on patient’s age and comorbidities
- Decision partially based on age/fitness and PSA (e.g., PSA 500 → CT TAP + bone scan rather than PET)
- For >T2 disease and PSA >10 → PSMA PET
- Struggle with capacity for bone scintigraphy – few patients with PSA <20
- PSMA PET CT even more tightly controlled – must meet strict staging parameters
- In some networks, bone scan and CT CAP easier to get than PSMA PET (postcode lottery)
- CT CAP often requested if high-risk patient referred to oncology
- PSMA PET often requested if high-risk patient considered for surgery
- Need up-to-date guidelines for PSMA PET-CT and whole-body MRI
- PSMA PET used for problem solving if CT/bone scan equivocal – limited availability and long delays
- Staging decided at MDT based on multiple factors
- PSMA PET for highly selected cohort
- Some patients get PSMA PET if doubtful/indeterminate lesions on CT or bone scan
- PSMA PET used for problem solving
- PSMA PET-CT done after MDT discussion if equivocal findings

**Free text responses to the question: Please add any additional comments you have related to scoring systems**

- Too many scoring systems, can make the report confusing for clinicians.
- PI-QUAL takes too long. Easier to say if the scan is diagnostic quality or not
- PI-QUAL I think is an unnecessary faff. Adds another score to cause confusion. Could be useful for auditing quality however. I do give a subjective quality assessment however. Eg satisfactory. If not satisfactory, I say why.
- Not needed by referrers in current practice
- We are discussing taking up precise at the moment but need all reporters to agree. We comment on quality which is based around the same parameters as PI-QUAL but feel this is unnecessary addition
- PI-FAB not applicable to our practice. Likert used instead of PI-RADS. Use descriptors of (poor) quality rather than PI-QUAL categorisation. Few post treatment-failure cases in our practice, so tend to be descriptive in reports
- If an MR is not good enough quality to diagnose it exclude significant PCa just say it in your report. Will only use PRECISE if Urologists ask for it
- Likert used routinely for patients without known PCa. I give an overall score, rather than per lesion, to simplify the message to the clinical team. Scan quality is described verbally, as are follow up scans for AS and post-treatment.
- Not familiar enough
- Not widely used in our MDT
- Aware of PRECISE and PI-QUAL but not routinely used by our radiology team so felt no need to cause any confusion to urologists who read the reports
- I believe the urologists are unfamiliar with the PI-QUAL scoring system. Although I do not provide an actual score, I do describe the deficiencies in the quality of the scan.
- too many scores may cause confusion
- Follow what the department generally uses
- I am using scoring systems that I am aware of
- Department has chosen not to use. Need to keep uniformity amongst reporters. Clearly communicating salient clinical points in a report or MDT is often sufficient rather than ascribing a score.
- Comment on Quality but I don't formally use PI-QUAL
- We audited our department for pi qual for all our scanner so do not routinely use
- Only so many hours in the day
- Planning to use PIQUAL
- Too complex
- Feel would confuse clinicians.
- Our surgeons understand PI-RADS. Our peripheral hospitals (3 other) understand it and use it. We don’t want to complicate across >I million patient catchment area.
- No demand from clinicians
- There is nothing wrong with PI-RADS and we have a lot of experience with it
- No added value.
- We have a proforma with a domain for image quality, which I use. In narrative reports, I will mention image quality if it is suboptimal or sub-diagnostic. I prefer to make a narrative technical note if needed. As for assessing for MRI progression, recurrence or post focal surveillance, again I prefer a narrative report, and I would often use a Likert score, which the clinicians recognise (I think they would mostly not recognise the other scoring systems). Ultimately, I want my report to reflect my view, which I can do without a scoring system. Perhaps they have more importance for a radiologist who is gaining experience.
- PI-QUAL and precise sound non-sensical to me.
- I prefer Likert to PI-RADS
- We don't do much focal therapy
- Very complex method of scoring
- I think PI-QUAL is a useful QA tool but probably not necessary for every scan. PI-FAB and PI-RR are only utilised in a small number of centres
- I regularly attend BSUR and attended the RCR accreditation course a few years ago when the teaching was very much to use all the clinical information available and thus use a Likert score. I have not been persuaded to change from this!
- Just use Likert and PRECISE to encompass the above.
- Follow our Cancer Network practice
- We use Likert rather than PI-RADS locally.
- Likert scores are able to take into account the other parameters outside imaging i.e. PSA/family history/PSA density to give a more clinical perspective on scoring.
- PI-QUAL - currently using to do an audit but no plans to use as part of reporting.
- PRECISE - not aware of any benefits of using it and none of my colleagues are using it and none of the clinicians are pushing us to use it.
- PI-QUAL does not add to improving local quality and more an audit tool. PRECISE too clunky and not clearly validated for altering outcome.
- I base my assessments on PI-RADS but use the Likert score to allow for clinical judgement.
- Aware of PRECISE but not routinely used in the reporting of surveillance imaging
- Only so much time in the day. We have a massive workload and are short of 2 uroradiologists. We keep MRI reports short and only include what clinicians need
- Likert only is standard practice where I trained and where I work
- Not something our institution uses. There are too many.
- PI-QUAL too complicated in my time constraints
- Don't like prescriptive rules of PI-RADS and we are a referral centre for focal therapy.
- Only Likert scores used in our organisation and region
- Usually give qualitative assessment of issues relating to scan quality (rather than formal PI-QUAL). Local ablative treatment not offered locally so PI-FAB not applicable to my patient population - would use if had eligible patients. PRECISE very useful to AS patients.
- Because the urologists prefer PI-RADS
- We use TARGET for post focal therapy cases
- PI-RR/PI-FAB - very few patients
- PI-QUAL - often detached from “clinical reality”. We only mention reduced quality if it has a direct impact on the ability of the study to rule in/out a tumour.
- Aware of the concepts of PI-QUAL, PRECISE, PI-RR which I try to incorporate into my reports, but think there's a limit to how many scoring systems clinical colleagues find useful.
- Clinicians do not necessarily find it useful
- Not routinely done in our center, maybe due to awareness amongst all reporters/urologists.

**Supplementary Table 1.** Full list of survey questions.

Legend: * Free text; % Binary choice; § Multiple choice; ^ Likert score

| **About your hospital** |
| --- |
| Which hospital/Trust do you represent? ^*^ |
| What category of hospital is this? ^§^ |
| How many radiologists report prostate MRI at your hospital? ^§^ |
| How many radiologists are regularly involved in a prostate MDT meeting at your hospital? ^§^ |
| Which Radiology Information System (RIS) code is used for prostate MRI at your hospital? ^*^ |
| **Prostate MRI protocols** |
| For suspected localised prostate cancer, do you use biparametric or multiparametric MRI as standard? ^%^ |
| If using multiparametric MRI, would you support a change to biparametric MRI? ^^^ |
| Please add any additional comments related to biparametric versus multiparametric MRI ^*^ |
| In addition to the sequences required for prostate MRI, do you perform additional sequences? ^§^ |
| What patient preparation steps are taken routinely for prostate MRI? ^§^ |
| **Suspected prostate cancer pathway** |
| In the setting of suspected prostate cancer, which is performed first, MRI or biopsy? ^%^ |
| Which biopsy technique is used? ^§^ |
| How are biopsy cores targeted towards lesions on MRI? ^§^ |
| Who performs prostate biopsy at your Trust? ^§^ |
| How is lesion location on MRI communicated to the person performing biopsy? ^§^ |
| Are cases with positive MRI but negative biopsies discussed in an MDT/post-biopsy meeting? ^§^ |
| In high or very high-risk prostate cancer, what would be your typical staging investigation? ^§^ |
| Please add any additional comments related to distant staging ^*^ |
| **Individual experience** |
| How many years of experience of prostate MRI reporting do you have art consultant level? ^§^ |
| Estimate many prostate MRI studies you have reported in total ^§^ |
| Estimate how many prostate MRI studies you have reported in the past year ^*^ |
| Have you attended a prostate MRI course in the past 3 years? ^%^ |
| How many prostate MRI courses have you attended in total? ^§^ |
| Rate your level of agreement with the following statements: ^^^ |
| Formal accreditation is necessary to ensure high quality prostate MRI reporting |
| I currently have plans to gain formal accreditation |
| If accreditation were to become mandatory, then I would look to gain accreditation |
| **In your prostate MRI reports** |
| Do you give a PI-RADS or Likert score? ^§^ |
| If giving a PI-RADS score, do you let clinical factors influence this? ^%^ |
| Which of the following scoring systems are using aware of? And using in routine practice? ^§^ |
| PI-RADS |
| PI-QUAL |
| PRECISE |
| PI-FAB |
| PI-RR |
| Please add any additional comments you have related to scoring systems ^*^ |
